# Antepartum Serum Lactate Dehydrogenase and Adverse Obstetric Outcomes in Preeclampsia: A Systematic Review of Prognostic Evidence From Low- and Middle-Income Countries

**DOI:** 10.7759/cureus.86496

**Published:** 2025-06-21

**Authors:** Ky D Nguyen, Anh TN Khuc, Vu A. Tran, Hieu S. Nguyen, Phong H. Nguyen, Anh TH. Nguyen

**Affiliations:** 1 General Medicine, Hanoi Medical University, Hanoi, VNM; 2 Obstestric and Gynecology, National Hospital of Obstetrics and Gynecology, Hanoi, VNM

**Keywords:** a systematic review, feto-maternal outcome, lactate dehydrogenase (ldh), low and middle country (lmic), preeclampsia-eclampsia

## Abstract

This systematic review evaluated the association between antepartum serum lactate dehydrogenase (LDH) levels and adverse maternal and perinatal outcomes in preeclamptic pregnancies in low- and middle-income countries (LMICs). A comprehensive search of PubMed, Embase, and Scopus identified 19 observational studies published between 2000 and 2025, comprising 5,039 pregnant women, including 3,782 preeclampsia diagnoses. Most studies were conducted in South Asia and the Middle East. Although LDH thresholds varied, a consistent trend was observed: higher LDH levels were associated with increased risk of maternal complications such as HELLP syndrome, disseminated intravascular coagulation, acute renal failure, and ICU admission. Perinatal complications, including stillbirth, intrauterine growth restriction, low Apgar scores, and neonatal intensive care unit (NICU) admission, were also more common at LDH levels >800 IU/L. For example, stillbirth occurred in up to 77.7% of cases in the highest LDH group compared to <3% in the lowest. Due to heterogeneity in cut-offs and outcome definitions, meta-analysis was not performed. Overall, elevated antepartum serum LDH appears to be a strong prognostic marker for severe outcomes in preeclamptic pregnancies and may aid clinical triage in LMICs. Further prospective studies are needed to establish standardized thresholds and validate their predictive utility.

## Introduction and background

Preeclampsia is a multisystem disorder that affects approximately 2-5% of pregnancies and remains a leading cause of maternal and perinatal morbidity and mortality worldwide [1]. The burden of disease is significantly higher in low- and middle-income countries (LMICs) [2], where adverse outcomes such as eclampsia, HELLP syndrome, pulmonary edema, and multiorgan failure in mothers, as well as intrauterine growth restriction (IUGR), preterm birth, or perinatal death in fetuses, are more prevalent. Early identification of high-risk pregnancies is critical for guiding management and allocating limited medical resources, particularly in LMICs, where healthcare capacity remains constrained. Without validated and widely adopted prognostic tools, lactate dehydrogenase (LDH) has emerged as a promising biochemical marker. LDH is an intracellular enzyme reflecting anaerobic metabolism secondary to placental ischemia, a key pathophysiological mechanism in preeclampsia. Preliminary studies have suggested that elevated serum LDH levels may be associated with an increased risk of maternal and perinatal complications [3-5]. However, the existing evidence remains scattered and has not been systematically synthesized. Therefore, we conducted this systematic review to synthesize and comprehensively assess the association between antepartum LDH levels and adverse obstetric outcomes among women with preeclampsia in low- and middle-income countries (LMICs).

## Review

Methodology

Data Sources and Search Strategy

This systematic review was not prospectively registered in PROSPERO due to the time constraints during its initial development. However, the review strictly adheres to PRISMA 2020 guidelines in its methodology and reporting. A comprehensive search strategy was developed and applied across four electronic databases: PubMed, Embase, and Scopus. The search covered the period from January 1, 2000, to April 30, 2025. Only articles published in English or Vietnamese were considered for inclusion.

The search strategy was constructed using combinations of controlled vocabulary (e.g., MeSH, Emtree terms) and free-text keywords related to “hypertensive disorders of pregnancy”, “preeclampsia”, and “lactate dehydrogenase” (LDH), along with terms referring to maternal and fetal outcomes. The whole search strategy for each database is available in the Supplementary Material. All records retrieved were imported into EndNote for de-duplication and then transferred to Rayyan AI for screening.

Eligibility Criteria and Study Selection

Studies were eligible for inclusion if they followed an observational design (cross-sectional, case-control, or cohort) and involved pregnant women diagnosed with preeclampsia. A key requirement was measuring maternal serum lactate dehydrogenase (LDH) levels during the antepartum period, specifically in the third trimester or within seven days before delivery. Furthermore, studies needed to report at least one adverse maternal or perinatal outcome and be published in either English or Vietnamese. We excluded reviews, editorials, commentaries, case reports, animal studies, and non-peer-reviewed sources. Two independent reviewers screened all titles and abstracts. Full-text screening was conducted for potentially eligible articles. Disagreements during selection were resolved through discussion or adjudicated by a third reviewer.

Quality Assessment and Data Extraction

Two reviewers independently assessed the quality of the included studies using the Joanna Briggs Institute (JBI) critical appraisal checklists for observational studies [6]. Any discrepancies were resolved through discussion and consensus. A standardized data extraction form, piloted on three studies, was used to collect the following information: study identifiers (author, year, country), population characteristics (type of preeclampsia, sample size), LDH measurement details (method, units, cut-offs, timing), reported maternal outcomes (e.g., ICU admission, maternal death, HELLP), and perinatal outcomes (e.g., preterm birth, NICU admission, low birth weight, intrauterine fetal demise (IUFD)) (Appendices: Tables 4-5). Effect estimates were also extracted when available (e.g., OR, RR, p-values). Data were entered into Excel spreadsheets and cross-checked for accuracy.

Data Synthesis and Analysis

Due to heterogeneity in LDH measurement thresholds and reporting formats across studies, no meta-analysis was conducted. Instead, results were synthesized narratively and tabulated by outcome category. We summarized trends across studies using LDH strata for maternal and fetal outcomes. Studies were grouped by outcome domain, and patterns were assessed qualitatively. Where applicable, thresholds for clinically significant LDH levels were highlighted based on the original study definitions. Due to limited homogeneity, no subgroup or sensitivity analyses were performed.

Result

Study Characteristics

**Figure 1:**
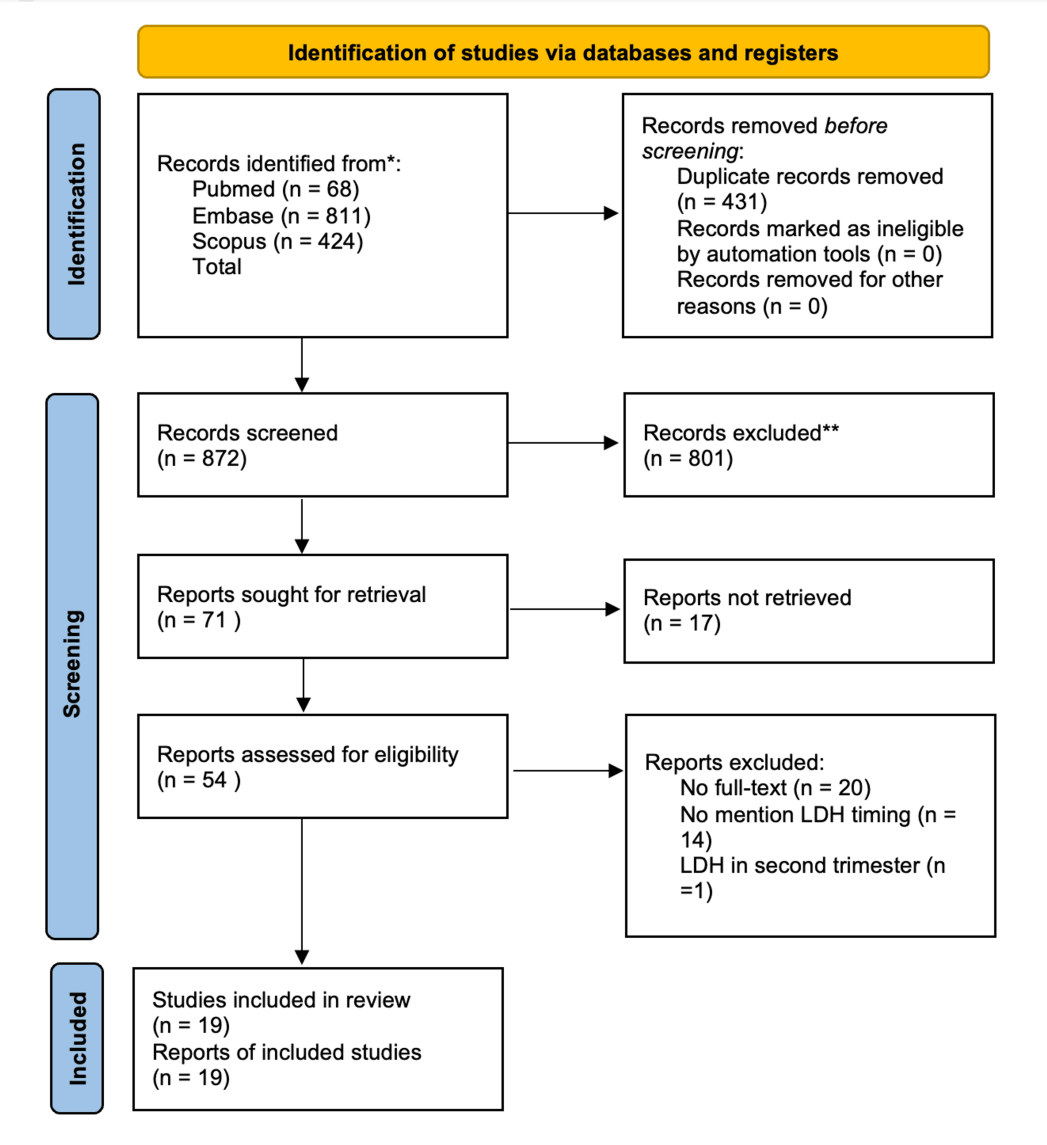
PRISMA flowchart

As shown in the PRISMA flow chart (Figure 1), an initial systematic search identified a total of 1,303 records from three databases: PubMed (n = 68), Embase (n = 811), and Scopus (n = 424). After removing 431 duplicates, 872 records remained for title and abstract screening. Of these, 801 records were excluded for not meeting the predefined eligibility criteria (e.g., irrelevant study population, no measurement of serum LDH, lack of obstetric outcome data, review articles, case reports, conference abstracts, or animal studies). A total of 71 full-text articles were assessed for eligibility. Among these, 17 were excluded due to inaccessible full text or failure to meet inclusion criteria after detailed evaluation. The remaining 54 studies were assessed in detail for methodological quality and relevance. Ultimately, 19 studies met all inclusion criteria and were included in the final qualitative synthesis of this systematic review.

The dataset included in this systematic review was derived from 19 observational studies comprising a cumulative sample of 5,039 pregnant women. Among them, 3,782 women were diagnosed with preeclampsia, while the remaining 1,257 participants served as the control group without preeclampsia. Most studies originated from research centers in South Asia (India, Pakistan, Nepal) and the Middle East (Iraq). The disease spectrum encompassed a wide range of hypertensive disorders in pregnancy, from mild preeclampsia to severe preeclampsia and eclampsia. Methodologically, the included studies employed various observational designs, including nine prospective cohort studies, five case-control studies, and five cross-sectional studies. Based on the JBI critical appraisal tool, most studies demonstrated moderate to high methodological quality, providing a reliable foundation for evidence synthesis. A key feature highlighted in Table 1 is the heterogeneity in LDH stratification across studies. A common approach was categorizing LDH levels into < 600 IU/L, 600-800 IU/L, and > 800 IU/L. Other studies applied alternative binary thresholds, such as > 214 IU/L, ≥ 300 IU/L, or ≥ 600 IU/L. This variability in LDH cut-off definitions underpinned the decision to adopt a narrative synthesis methodology, enabling qualitative comparisons across differently defined LDH strata.

**Table 1:** Characteristics of Included Studies JBI: Joanna Briggs Institute

Author, Year	Country	LDH Threshold Value(IU/L)	Study Population Characteristics and Sample Size	Study Design	JBI Appraisal
Noor et al. 2020 [7]	India	<600, 600-800, >800	<600 IU/L: 116, 600-800 IU/L: 13, >800 IU/L: 21	Observational prospective study	Moderate
Dave et al. 2016 [8]	India	<600, 600-800, >800	Control: 100, severe preeclampsia: 32, eclampsia: 68	Prospective observational study	High
Jaiswar et al. 2011 [9]	India	<600, 600-800, >800	Normal pregnant women: 39, mild preeclampsia: 35, severe preeclampsia: 36, eclampsia: 36	Prospective comparative study	High
Nasir et al. 2025 [10]	Iraq	135-214,>214	Mild preeclampsia: 50, severe preeclampsia: 50, control group: 50	Prospective case-control study	High
Hak et al. 2015 [11]	India	<600,600-800, >800	Controls: 100, mild preeclampsia: 33, severe preeclampsia: 35, eclampsia: 32	Case-control study	Moderate
Padmavathi et al. 2019 [12]	India	<600, 600-800, >800	Normal pregnant women: 35, mild preeclampsia: 35, severe preeclampsia: 35, eclampsia: 35	Prospective comparative study	Moderate
Dasarwar and Patil 2019 [13]	India	<600, 600-800, >800	Mild preeclampsia: 46, severe preeclampsia: 38	Prospective study	Moderate
Reddy et al. 2023 [14]	India	<600, 600-800, >800	Normotensive group: 115, preeclamptic-eclamptic group: 115, mild preeclampsia: 60, severe preeclampsia: 40, eclampsia: 15	Observational cohort study	High
Devika et al. 2021 [15]	India	<600, 600-800, >800	Normal pregnant women: 39, mild preeclampsia: 35, severe preeclampsia: 36, eclampsia: 36	Prospective comparative study	High
Garg et al. 2019 [16]	India	<600, 600-800, >800	Mild preeclampsia: 81, severe preeclampsia: 51, eclampsia: 8	Prospective study	High
Kumari et al. 2024 [17]	India	<600, 600-800, >800	Normotensive controls: 223, mild preeclampsia: 142, severe preeclampsia: 81	Case-control study	High
Vyas et al. 2021 [18]	India	<600, >600	LDH <600 IU/L: 96, LDH >600 IU/L: 19	Prospective study	Moderate
Moharana et al. 2023 [19]	India	<300, ≥300, 300-600, 600-800, >800	Patients with normal LDH and uric acid levels: 300, patients with abnormal LDH and uric acid levels: 900	Cross-sectional study	High
Prajapati et al. 2021 [20]	Nepal	<600, 600-800, >800	Gestational hypertension: 71, mild preeclampsia: 49, severe preeclampsia: 41, eclampsia: 19, LDH <600 IU/L: 99, LDH 600-800 IU/L: 48, LDH >800 IU/L: 33	Hospital-based observational descriptive study	Moderate
Salman and Rafiq 2022 [21]	Pakistan	<600, ≥ 600	LDH <600 IU/L: 101 patients, LDH ≥ 600 IU/L: 101 patients	Prospective cohort study	High
Khillan and Kaur 2018 [22]	India	<600, 600-800, >800	Normotensive: 50, mild preeclampsia: 50, severe preeclampsia: 50, eclampsia: 50	Prospective observational study	High
Dev and Hemalatha 2019 [23]	India	<600, 600-800, >800	Normotensive pregnant women: 60, mild preeclampsia: 60, severe preeclampsia: 60, eclampsia: 30	Prospective comparative study	High
Deshmukh et al. 2020 [24]	India	<600, 600-800, >800	Control group: 100, case group: 100, non-severe preeclampsia: 33, severe preeclampsia: 30, eclampsia: 37	Prospective comparative case-control study	High
Mary et al. 2017 [25]	India	<600, 600-800, >800	Healthy normotensive controls: 200, mild preeclampsia: 121, severe preeclampsia: 79	Cross-sectional case-control study	High

Maternal Complications

From a clinical obstetric perspective, Table 2 demonstrates that elevated antepartum serum LDH levels are strongly associated with the occurrence and severity of maternal complications in preeclamptic pregnancies. Among the 3-tiered LDH stratification-<600 IU/L, 600-800 IU/L, and >800 IU/L-the- the>800 IU/L group consistently showed the highest rates of adverse outcomes.

**Table 2:** Association Between Antepartum LDH Levels and Adverse Maternal Outcomes in Preeclampsia LDH: lactate dehydrogenase

Adverse Maternal Outcome	No. of Studies	LDH Threshold (IU/L)	Complication Rate (Min-Max)	Summary of Key Findings on the Association
HELLP syndrome	15 [7,9-18,20,23-25]	<600	0.7% - 2.0%	
		600-800	2.5% - 18.2%	Studies show a wide range of HELLP rates in the intermediate group.
		>800	6.9% - 77.8%	The high LDH group has the highest HELLP rate, reflecting the severity of this complication; the mean LDH among HELLP patients is 1303.6 ± 456.0 IU/L.
Disseminated Intravascular Coagulation (DIC)	19 [7-25]	<600	0% - 1.3%	DIC is very rare or absent in the low LDH group.
		600-800	1.6% - 30.6%	The DIC rate starts to increase in the medium LDH group.
		>800	4.8% - 70.4%	DIC rises sharply with higher LDH, particularly > 800 IU/L; for example, 70.4 % of cases in the > 800 IU/L group had DIC.
Acute Renal Failure	11 [8-9,12-15,17,20,22,24-25]	<600	0% - 1.7%	Acute renal failure risk is low or absent in the low LDH group.
		600-800	3.2% - 12.1%	ARF risk begins to rise in the medium LDH group.
		>800	5.5% - 51.9%	ARF risk escalates markedly at LDH > 800 IU/L; 51.9 % of women in this group developed ARF.
Pulmonary edema	14 [7-11,13-14,16,18,20,22-25]	<600	1.1% - 7.8%	The frequency of pulmonary edema was comparable between the two groups.
		600-800	1.6% - 7.7%	
		>800	12.8% - 38.1%	Pulmonary edema is a complication that increases with higher LDH levels. For example, 38.1% of cases in the high LDH group had pulmonary edema.
Intracerebral hemorrhage	12 [7-12,14-17,22,25]	<600	0%	The incidence of intracerebral hemorrhage is very low or absent in the low LDH group.
		600-800	0% - 7.7%	Some studies have reported intracerebral hemorrhage in the medium LDH group.
		>800	3.1% - 18.5%	The incidence of intracerebral hemorrhage increased significantly in the high LDH group, particularly among those with LDH levels >800 IU/L. For example, 18.5% of cases in the high LDH group had intracerebral hemorrhage.
Eclampsia	16 [7-11,13-20,22,24,25]	<600	0.7% - 5.1%	
		600-800	4.1 – 11.1%	The incidence of eclampsia begins to emerge and increases in the medium LDH group.
		>800	3.4% - 40.6%	Preeclampsia is more likely to progress to eclampsia in women with elevated LDH levels.
Placental abruption	14 [8-9,11-17,20,22-25]	<600	0.9% - 4.1%	The incidence of placental abruption is low or absent in the low LDH group.
		600-800	3.2% - 24.2%	The incidence of placental abruption begins to increase in the medium LDH group.
		>800	6.9% - 66.7%	The incidence of placental abruption increases progressively with higher LDH levels, indicating a clear association. For example, 66.7% of cases in the high LDH group experienced placental abruption.
Maternal ICU admission	5 [7-8,17,20-21]	<600	0%	The rate of maternal ICU admission is absent in the low LDH group.
		600-800	0-22.7%	The rate of maternal ICU admission begins to increase in the medium LDH group.
		>800	14.7-77.8%	The rate of maternal ICU admission increases significantly with higher serum LDH levels. Specifically, 77.8% of cases in the high LDH group required ICU admission.
Maternal death	9 [7-9,13-14,18,20,22,24]	<600	0%	
		600-800	0%	
		>800	0-22.2%	Elevated LDH levels are significantly associated with maternal mortality. In several studies, all cases of maternal death occurred in the group with LDH levels >800 IU/L.

HELLP syndrome, a severe manifestation of endothelial dysfunction and microangiopathic hemolysis, occurred in 25-77.8% of patients with LDH >800 IU/L across studies [8,9,22]. For example, Jaiswar et at reported HELLP in 77.8% of patients with LDH >800 IU/L, while none of the patients with LDH <600 IU/L developed the syndrome [9]. This stark contrast reinforces the discriminative capacity of LDH.

Disseminated Intravascular Coagulation (DIC) showed a similarly alarming trend. In the >800 IU/L group, the incidence ranged from 28.1% to 71.4%, whereas DIC was rare (≤2%) or completely absent in patients with LDH <600 IU/L [7,22].

Acute renal failure (ARF), a common and life-threatening complication of severe preeclampsia, was noted in 29.6% to 51.9% of patients with LDH >800 IU/L, while remaining below 5% in the <600 IU/L group. Pulmonary edema, often a direct consequence of increased capillary permeability, showed a striking increase with LDH elevation. It was reported in 15-33.3% of patients with LDH >800 IU/L, but rarely (0-2.9%) in those with LDH <600 IU/L [8,21].

Maternal ICU admission- an integrated indicator of clinical severity- was consistently higher in the >800 IU/L group. For instance, Kumari et al. reported ICU admission in 40.7% of patients with LDH >800 IU/L compared to 3.7% in the 600-800 IU/L group and none in the <600 IU/L group [17].

Perhaps most critically, maternal mortality was reported in 22.2% of patients with LDH >800 IU/L, while there were no maternal deaths in the LDH <600 IU/L and 600-800 IU/L groups across all studies [13].

This progressive increase in complication rates not only highlights a dose-response relationship between serum LDH and adverse maternal outcomes but also suggests the potential of LDH as a clinically actionable biomarker. The low incidence of severe complications in the <600 IU/L group suggests a possible negative predictive value for LDH at this threshold, whereas levels exceeding 800 IU/L may warrant immediate intervention and tertiary care referral.

These findings emphasize that LDH is more than a metabolic by-product - it is a surrogate for the severity of the underlying pathophysiology in preeclampsia. In low-resource settings, serum LDH could be a valuable triage tool to guide obstetric decision-making and optimize maternal outcomes. 

Fetal Complications

Adverse fetal and neonatal outcomes also showed a clear association with maternal LDH levels, as detailed in Table 3.

**Table 3:** Association Between Antepartum LDH Levels and Adverse Perinatal Outcomes in Preeclampsia

Adverse Perinatal Outcomes	No. of Studies Reporting	Relevant LDH Threshold (IU/L)	Complication Rate (Min-Max)	Summary of Key Findings
Intrauterine Growth Restriction (IUGR)	5 [14,17,18,20,24]	≥600, >800	4% (for LDH levels > normal) to 48.65% (>800 IU/L)	High LDH levels correlate with a higher incidence of IUGR. Specifically, the rate of IUGR in the LDH >800 IU/L group can be as high as 48.65%. Other studies also note a significant increase in IUGR as maternal LDH increases.
Intrauterine Fetal Death	15 [7–9,11,14–17,19–24]	>800	4.4% (>800 IU/L) to 77.7% (>800 IU/L)	Elevated serum LDH levels (especially >800 IU/L) are strongly and statistically significantly associated with a higher risk of stillbirth/intrauterine fetal death. For example, the stillbirth rate can be as high as 60% or 77.7% in the LDH >800 IU/L group. Many other studies also confirm this association.
Neonatal Intensive Care Unit admission	5 [10,13,17,23,24]	<600,600-800, >800	7.75% (<600 IU/L) to 100% (>800 IU/L)	The rate of newborns requiring NICU admission increases significantly as maternal LDH levels rise. Cases with LDH >800 IU/L have the highest NICU admission rate, for example, 57.14% or even 100% in some studies. This association is statistically significant in the majority of studies.
Low Apgar Score	6 [7,9,13,15,18,24]	>800	A trend from a low incidence of poor scores to an overall lower average score	High LDH levels correlate with significantly lower Apgar scores in newborns at both 1 and 5 minutes after birth. Some studies note that this association is statistically significant.
Preterm birth/Gestational age	10 [9–12,15,20–24]	Elevated LDH levels are correlated with decreased gestational age	The average gestational age decreases from 38.4 weeks (<600 IU/L) to 33.25 weeks (>800 IU/L)	Higher LDH levels are significantly associated with a lower mean gestational age at birth, indicating a higher rate of preterm birth in cases with elevated LDH. For example, the mean gestational age can decrease from ~38 weeks in the low LDH group down to ~33-35 weeks in the high LDH group (>800 IU/L).
Perinatal Mortality	13 [7,9–12,15–17,20,22–25]	>800	0.9% (<600 IU/L) to 77.7% (>800 IU/L)	The perinatal mortality rate is highest in the LDH >800 IU/L group, with a significant increase compared to lower LDH groups. This rate can be as high as 77.7% in some studies. This association is confirmed to be statistically significant in many studies.
Respiratory Distress Syndrome (RDS/ARDS)	7 [7,9–12,15,24]	>800; High LDH	10.4% (>800 IU/L) to 33.3% (High LDH)	RDS/ARDS is more common in newborns of mothers with higher LDH levels, for example, 33.3% in the high LDH group compared to 12% in the normal LDH group.
Hypoxic Ischemic Encephalopathy (HIE)/Birth Asphyxia	2 [14,19]	>800	0.2% (<600 IU/L) to 3.7% (>800 IU/L)	High LDH levels correlate with a higher incidence of HIE/birth asphyxia, especially in the LDH >800 IU/L group.
Meconium Aspiration Syndrome	2 [9,15]	>800	3.4% (>800 IU/L)	This is a complication noted to be associated with higher LDH levels.

IUGR was documented in nine studies and showed a marked escalation with increasing LDH. The incidence ranged from 4.1% in the <600 IU/L group to 48.7% in the >800 IU/L group [17,21]. Devika et al. found IUGR in 45.9% of neonates whose mothers had LDH >800 IU/L, compared to only 7.2% with LDH <600 IU/L - a sevenfold increase [15].

Stillbirths (IUFD) exhibited one of the most dramatic differentials. Among women with LDH <600 IU/L, stillbirth rates ranged from 0% to 2.7%, whereas in the >800 IU/L group, rates reached 77.7% in studies by Noor and 59.3% in Salman et al. [7,21]. These figures illustrate a nearly 30-fold increase in fetal death risk with critical LDH elevation.

Preterm birth was another consistently reported outcome. In 12 studies, the mean gestational age at delivery decreased from 38.4 weeks in the LDH <600 IU/L group to as low as 33.2 weeks in those with LDH >800 IU/L [17,22]. This 5-week gap may reflect both spontaneous and iatrogenic preterm deliveries triggered by worsening maternal disease.

NICU admissions further illustrate this risk. While NICU utilization was minimal (<10%) among infants born to mothers with LDH <600 IU/L, it increased to 52.2-100% in the LDH >800 IU/L group across multiple studies.

Low Apgar scores (≤7 at 1 and 5 minutes) were significantly more prevalent in neonates born to mothers with LDH >800 IU/L. Noor et al. noted a low 1-minute Apgar in 70.3% of this group, compared to just 3.7% in the <600 IU/L group [7]. Similarly, Salman et al. found 5-minute Apgar ≤7 in 62.9% versus 1.7%, respectively [21].

Additional complications such as respiratory distress syndrome (RDS), meconium aspiration syndrome (MAS), hypoxic ischemic encephalopathy (HIE), and very low birth weight (<2,000 g) were disproportionately concentrated in the >800 IU/L cohort, with reported rates ranging from 20-50%.

These findings strongly support serum LDH as a maternal disease marker and a sentinel fetal indicator. Elevated LDH reflects placental dysfunction and systemic endothelial damage, which compromise fetal perfusion, growth, and oxygenation. Therefore, LDH should be integrated into perinatal risk models, especially in LMIC settings, where low-cost markers are critically needed to guide obstetric intervention. 

Discussion

Key Findings

Our systematic review confirms a strong association between elevated antepartum LDH levels and adverse outcomes in preeclamptic pregnancies. Across these diverse LMIC cohorts, higher maternal LDH consistently correlated with more severe maternal complications - including HELLP syndrome, DIC, acute renal failure, pulmonary edema, and even maternal death - especially pronounced when LDH exceeded 800 IU/L. Likewise, fetal and neonatal outcomes worsened with rising LDH: mothers with LDH in the >800 IU/L range had dramatically higher rates of IUGR, stillbirth, preterm delivery, low Apgar scores, and NICU admissions compared to those with LDH <600 IU/L. Notably, several studies reported no maternal deaths in women whose LDH remained <600 IU/L, whereas all observed maternal fatalities occurred in the highest LDH category. Overall, our findings highlight a clear dose-response relationship - women with critically elevated LDH levels before delivery face the most significant risk of severe preeclampsia-related complications.

Strengths and limitations

Strengths

This review is the first to comprehensively synthesize evidence on LDH as a prognostic marker in preeclampsia, focusing on low - and middle-income countries. We employed a broad, systematic search (2000-2025) and adhered to PRISMA guidelines, enhancing the rigor and reproducibility of our findings. Inclusion of 19 observational studies from multiple regions (South Asia, Middle East, etc.) and a sizable pooled sample improves the generalizability of results and allows us to discern consistent trends despite heterogeneous settings. We also conducted formal quality appraisal (JBI checklists), and most studies were of moderate-to-high quality, lending credibility to the aggregated evidence. Importantly, by aggregating data across various LDH thresholds, our review was able to demonstrate the robustness of the LDH-outcome association (e.g., the stepwise increase in complications from <600 to 600-800 to >800 IU/L), which might not be apparent in any single study.

Limitations

Several limitations must be acknowledged. First, substantial heterogeneity in LDH measurement timing, cut-off values, and outcome definitions across studies precluded a meta-analytical pooling of effect sizes - our synthesis is mainly qualitative. Many included studies stratified LDH differently (e.g., some used 600/800 IU/L tiers, others used alternate cut-offs), and the definition of “adverse outcome” was not uniform, complicating direct comparisons. Second, the evidence base consisted of observational hospital-based studies (prospective cohorts, case-controls, etc.), often from single tertiary centers. These designs are prone to selection bias and confounding; for instance, high LDH may reflect more severe underlying disease rather than independently causing poor outcomes. Third, by focusing on LMIC populations, our review addresses high-burden settings but may limit generalizability to high-income countries - differences in healthcare infrastructure could modulate the absolute risks associated with a given LDH level. We also recognize that publication bias is possible (most included studies reported positive correlations), and some relevant data (mainly unpublished or non-English studies) might have been missed. Finally, the review was not registered in PROSPERO and relied on published data; ongoing or future extensive studies might further inform the prognostic value of LDH. These limitations suggest caution in interpreting LDH as a standalone predictor and reinforce the need for more standardized research on this biomarker.

Interpretation

Our findings align closely with a growing global literature linking elevated LDH to preeclampsia severity and complications. Numerous studies in resource-limited settings have reported similar patterns. For example, an extensive Indian survey of 1,200 women found that patients with LDH >800 IU/L experienced significantly higher rates of maternal and fetal complications compared to those with lower LDH [19]. In Latin America, Moya-Salazar et al. documented that over 70% of preeclamptic women in a Peruvian cohort had elevated LDH; notably, LDH rose in tandem with disease severity, a trend consistent with reports from South Asia [26]. Even an extensive multi-year analysis in Mexico concluded that serum LDH is a reliable marker of preeclampsia severity and is associated with adverse maternal outcomes [27]. These studies from Asia, Africa, and Latin America reinforce a consensus that LDH elevation reflects the degree of endothelial damage and tissue injury in preeclampsia, thereby serving as a proxy for the risk of complications.

Notably, the prognostic significance of LDH is apparent even when considering differences between low-resource and high-resource settings. The absolute rates of adverse outcomes can vary. For instance, maternal mortality due to preeclampsia is about seven times higher in developing regions than in developed countries (2.8% vs 0.4% according to WHO) [28]. This disparity is mainly attributable to obstetric intervention and critical care support. Nonetheless, the underlying relationship between LDH and disease severity seems biologically consistent across populations. In fact, in high-income settings, LDH is an established part of the clinical picture of severe preeclampsia; for example, hemolysis in HELLP syndrome is defined in part by elevated LDH (typically >600 IU/L). Our results underscore that a readily available test like LDH can have prognostic utility across diverse healthcare contexts. In resource-rich countries, aggressive management often prevents many LDH-elevated cases from progressing to fatal outcomes - yet the trend of worse outcomes with higher LDH remains evident. By contrast, in many LMIC hospitals where advanced assays (e.g., placental growth factor or sFlt-1) are not routinely available, LDH may serve as a convenient, low-cost risk stratification tool to flag high-risk preeclamptic patients. Ultimately, while LDH alone is not a perfect predictor, our review’s concordance with international studies bolsters the view that it is a meaningful indicator of preeclampsia severity and can help inform clinical decision-making. Further research is warranted to refine its prognostic thresholds and to integrate LDH with other markers. Still, the global evidence base suggests that this enzyme’s elevation should not be overlooked when managing preeclampsia.

## Conclusions

Despite growing interest in the use of biomarkers for risk stratification, preeclampsia continues to pose a substantial threat to maternal and perinatal health, particularly in LMICs. This systematic review highlights a consistent association between elevated antepartum serum LDH levels and an increased risk of adverse obstetric outcomes in women with preeclampsia. However, considerable variability in LDH thresholds, measurement timing, and outcome definitions limits its applicability in routine clinical settings. While LDH may serve as a readily available and low-cost biochemical marker in resource-constrained environments, current evidence is insufficient to recommend its standalone use as a prognostic tool. Further prospective, standardized, and adequately powered studies are needed to validate the predictive value of LDH and define optimal cut-offs that can guide clinical decision-making in preeclampsia management.

## Appendices

Systematic Review Protocol (PRISMA-P Format)

1. Title

Antepartum Serum Lactate Dehydrogenase and Adverse Obstetric Outcomes in Preeclampsia: A Systematic Review of Prognostic Evidence from Low- and Middle-Income Countries

2. Registration

This protocol will not be registered on PROSPERO due to author preference and time constraints. However, the complete protocol is transparently documented and available upon request.

3. Authors

Nguyen Dinh Ky, Khuc Thi Ngoc Anh, Tran Anh Vu, Nguyen Sy Hieu, Nguyen Han Phong, Nguyen Thi Huyen Anh

Affiliation: Hanoi Medical University, National Hospital of Obstetrics and Gynecology

Corresponding Author: Nguyen Thi Huyen Anh, MD, PhD https://www.researchgate.net/profile/Nguyen-Thi-Huyen-Anh 

4. Amendments

Any amendments to this protocol will be documented with a rationale and date and updated in the PROSPERO record.

5. Support

This review receives no specific external funding and is supported by internal institutional resources.

6. Roles and contributions

All listed authors contributed to the development of the protocol. Nguyen Dinh Ky is the guarantor. All team members will participate in screening, data extraction, and synthesis.

7. Rationale

Hypertensive disorders of pregnancy (HDP) are major contributors to maternal and perinatal morbidity and mortality globally. Lactate dehydrogenase (LDH) is an intracellular enzyme released during tissue damage and cellular stress, commonly elevated in preeclampsia. While numerous studies have investigated the relationship between elevated maternal serum LDH levels and obstetric outcomes, no comprehensive synthesis exists. This systematic review will evaluate and consolidate available evidence to clarify the prognostic value of maternal LDH levels in predicting adverse maternal and fetal outcomes in HDP.

8. Objectives

To evaluate the association between elevated maternal serum LDH levels and adverse maternal and perinatal outcomes in pregnant women with hypertensive disorders of pregnancy.

9. Eligibility criteria

Population: Pregnant women diagnosed with hypertensive disorders of pregnancy (gestational hypertension, preeclampsia, chronic hypertension with superimposed preeclampsia). Exposure: Elevated maternal serum LDH levels measured during the third trimester (≥28 weeks of gestation) or within 7 days before delivery, using any standard laboratory method.. Comparator: Normal or lower maternal LDH levels. Outcomes: Adverse maternal outcomes (e.g., ICU admission, maternal death, postpartum hemorrhage) and perinatal outcomes (e.g., preterm birth, low birth weight, intrauterine fetal death, low Apgar score, neonatal death).

Study types: Observational studies (cohort, case-control, cross-sectional). Language: English and Vietnamese. Exclusions: Reviews, case reports, editorials, non-peer-reviewed sources, animal studies.

10. Information sources

Databases: PubMed, Embase, Scopus. Additional sources: Forward/backward citation tracking and expert consultation as necessary.

11. Search strategy

A comprehensive search strategy will be applied using controlled vocabulary (e.g., MeSH, Emtree) and free-text keywords. The search will combine terms related to hypertensive disorders of pregnancy, LDH, and maternal/fetal outcomes. Strategies will be customized for each database and reviewed using the PRESS checklist. The search period is from January 1, 2000, to May 15, 2025.

12. Study records

Data management: EndNote will be used for reference management and deduplication; Rayyan AI will be used for title/abstract and full-text screening. Selection process: Two independent reviewers will screen records. Discrepancies will be resolved by discussion or a third reviewer. Data extraction: A standardized extraction form will be piloted and used by two independent reviewers.

13. Data items

Study characteristics (author, year, country, study design, sample size), participant characteristics, LDH measurement details (timing, method, cutoffs), obstetric outcomes (maternal and fetal), effect estimates (OR, RR, mean difference, p-values), and risk of bias indicators.

14. Outcomes and prioritization

Primary outcomes: maternal death, ICU admission, postpartum hemorrhage. 

Secondary outcomes: Preterm birth, low birth weight, low Apgar scores, intrauterine fetal death, neonatal death.

Exploratory: LDH thresholds for severity prediction.

15. Risk of bias in individual studies

Two reviewers will assess risk of bias using validated tools such as the Newcastle-Ottawa Scale (NOS) and JBI checklists for observational studies. Disagreements will be resolved through consensus or a third reviewer.

16. Data synthesis

Where feasible, meta-analysis will be conducted for outcomes reported by ≥2 studies using comparable effect measures. Random-effects models will be applied due to expected heterogeneity. Forest plots and I2 statistics will be used. Narrative synthesis and subgroup analysis will be conducted where meta-analysis is not feasible.

17. Meta-bias(es)

Publication bias will be assessed using funnel plots and Egger’s test if ≥10 studies are available for a given outcome.

18. Confidence in cumulative evidence

The quality and consistency of the evidence will be evaluated using GRADE criteria across outcomes, including risk of bias, consistency, directness, precision, and publication bias.

19. Ethics and dissemination

Ethical approval is not required because this systematic review is based on previously published studies and involves no direct contact with human subjects. The findings will be submitted for publication in a peer-reviewed journal and disseminated through academic conferences, institutional repositories, and professional networks to inform clinical practice and future research.

**Table 4:** Summary of Outcomes and Definitions

Outcome Category	Definition	Measurement/Extraction Approach
Maternal Death	Death of the mother during pregnancy, delivery, or postpartum	Binary outcome, extracted from study results
ICU Admission	Admission of pregnant woman to intensive care unit	Reported incidence based on LDH group comparison
Postpartum Hemorrhage	Excessive bleeding (>500ml vaginal or >1000ml cesarean) post-delivery	Extracted if LDH used as predictive marker
Preterm Birth	Delivery before 37 completed weeks of gestation	Extracted as percentage or frequency in LDH strata
Low Birth Weight	Birth weight <2500g	Extracted from fetal outcomes stratified by LDH
Intrauterine Fetal Death	Fetal death after 20 weeks of gestation	Binary outcome extracted if stratified by LDH
Low Apgar Score	Apgar score <7 at 1 or 5 minutes	Recorded per LDH level groups
Neonatal Death	Death of neonate within 28 days of life	Binary outcome related to maternal LDH
LDH Thresholds	Serum LDH cut-offs used to define risk (e.g., >600 IU/L, >800 IU/L)	Document exact cut-off values, categories, and their clinical relevance

**Table 5:** Data extraction field

Field	Description
Study Characteristics	Author(s), year of publication, country, journal, study design, sample size
Population Details	Type of hypertensive disorder (preeclampsia, gestational hypertension, chronic hypertension)
LDH Measurement Info	LDH level (mean, median, range), cut-off points, units, assay method
Maternal Outcomes	ICU admission, postpartum hemorrhage, maternal death
Fetal Outcomes	Preterm birth, low birth weight, Apgar score, IUFD, neonatal death
Statistical Results	p-values, adjusted OR/RR, confidence intervals, correlation coefficients
Study Quality/Bias	JBI scores
Funding and Conflicts	Whether study reported funding source or conflicts of interest
Notes/Comments	Additional reviewer comments, unusual findings, or methodological limitations
